# Supplementary material for: Transforming trade for vaccine equity: Policy gaps and barriers
Source: PLOS Glob Public Health. 2025 Jun 16;5(6):e0004012. doi: 10.1371/journal.pgph.0004012 (PMC12169585; doi:10.1371/journal.pgph.0004012)
Supplement: S1 Appendix — (DOCX) [file pgph.0004012.s001.docx]

**S1 Appendix:** description of organisations from which sources were drawn [see included document: Unformatted Tables VE]

| **Organisation** | **Type** | **Role** | **Documents** |
| --- | --- | --- | --- |
| UN Conference on Trade and Development (UNCTAD) | International multilateral organisation | “Supporting developing countries to access benefits of a globalised society.” | - Policy guides & tools - Market summaries - Online course handbook |
| World Trade Organisation (WTO) | International organisation run by member governments | “Operates a global system of trade rules, acts as a forum for negotiating trade agreements, settles trade disputes between its members and supports the needs of developing countries.” | - Trade reports - Trade obstruction lists - Policy guides - Policy summaries - Rules and protocols - Meeting memoranda - Books and chapters |
| OECD | International organisation | “Evidence-based international standards [solutions, and policies for] a range of social, economic and environmental challenges.” | - Policy advisories - Policy summaries - Trade reports - Conference proceedings - Economic assessments |
| European Union (EU) | Union of nations    Trade bloc | “Uphold and promote member nations values and interests,” “enhance economic cohesion and solidarity” among them “and promote fair and free trade” for “sustainable development within the wider world.” | - Trade reports - Policy guides - Laws - Partnership documents |
| World Health Organisation (WHO) | International organisation    United Nations agency | “Leads global efforts to expand universal health coverage,” directing and coordinating emergency responses, and “promoting healthier lives […] guided by science”. | - Policy guides - Roadmaps, reference guides and systematic reviews - National, regional, and global strategy documents - Regional consultations - Commission reports Director-General reports - Legal recommendations - Books and chapters |
| World Intellectual Property Organisation | Multilateral Organisation | “Global forum for intellectual property policy, services, information and cooperation.” | - FTA briefings - FTA negotiations |
| World Bank | Development Bank | “A family of five international organizations that make leveraged loans to developing countries” with the “twin goals of ending extreme poverty and building shared prosperity.” | - Review of trade agreements - Country partnership strategies - Pharmaceutical industry briefings |
| Other UN bodies – UNDP, UNESCAP, HDRO, IPCIG, UNU | International multilateral organisation | “Support countries in achieving the SDGs through integrated solutions.” | - Competition law report - Conference proceedings - Policy reports - Finance reports |
| African Union | Union of nations    Trade bloc | “Continental body consisting of the 55 member states that make up the countries of the African Continent.” | - International relations report - Negotiation proceedings |
| Oxfam International | Charity | “Confederation of independent non-governmental organizations came together in 1995 to share knowledge and resources and combine their efforts in the fight against poverty and injustice” | - Research papers - Trade reports - Critique of pharmaceutical industry |
| Asian Development Bank | Development Bank | Aims to eradicate extreme poverty by providing loans, assistance, grants, and equity investments, as well as facilitating policymaking. | - Trade facilitation report - Governance review |
| European Medicines Agency | Medicines Regulatory Authority | “Evaluation and supervision of medicines, for the benefit of public and animal health in the European Union (EU).” | - Pharmacovigilance reports |
| House of Commons Library | Government | “Provide a range of research and information services for members of the British Parliament.” | - Parliamentary research briefing |
| European Centre for International Political Economy (ECIPE) | Think Tank | “Independent and non-profit policy research think tank dedicated to trade policy and other international economic policy issues of importance to Europe” | - Book chapter |
| The Global Fund to Fight AIDS, Tuberculosis and Malaria | Non-profit organisation;  NGO | “Raise and invest US$4 billion a year to fight AIDS, tuberculosis and malaria”, “challenge injustice and strengthen health systems” | - Initiative - Analytical report |
| Independent Panel for Pandemic Preparedness and Response | Body of the WHO (from WHA 73.1) | Provide an “evidence-based path for the future, grounded in lessons of the present and the past to ensure countries and global institutions, including specifically WHO, effectively address health threats.” | - Legal recommendations - Background research papers |
| New Markets Lab | Policy Hackathon | “A law and development center focused on integrating economic and social considerations into the design and implementation of law and regulation” | - Policy report |
| International Centre for Research and Agroforestry | Think Tank | “Develop knowledge practices to ensure food security and environmental sustainability.” | - Book on Value Chain Development |
| South African Institute of International Affairs | Think Tank | “Independent public policy think tank advancing a well-governed, peaceful, economically sustainable and globally engaged Africa.” | - Research paper - Policy reports |
| Rajaratnam School of International Studies | Think Tank | “Strategic Studies, International Relations, International Political Economy, and Asian Studies.” | - International relations report - Reviews |
| Montreal Economic Institute | Think Tank | “[Promoting] economic liberalism education of the general public and […] efficient public policies in Quebec and Canada.” | - Policy reports |
| International Institute for Sustainable Development | Non-Governmental Organisation | “Recommends on policies regarding international trade and investment, economic policy, management of natural and social capital, and information technologies” | - Trade review of SDGs |
| ActionAid Australia | NGO | “We empower women on the frontlines of injustice to work together and transform their communities.” | - Research paper |
| Wilson Centre | Think Tank | “Non-partisan policy forum [chartered by the US congress] for tackling global issues through independent research and open dialogue to inform actionable ideas.” | - Trade outlook report |
| European Economic and Social Committee | Multilateral Organisation | “Contributes to strengthening the democratic legitimacy and effectiveness of the European Union by enabling civil society organisations from the Member States to express their views at European level” | - Report - Impact assessments |
| Asian-Pacific Economic Cooperation | Multilateral Organisation | “Inter-governmental forum for 21 member economies in the Pacific Rim that promotes free trade throughout the Asia-Pacific region.” | - Policy report |
| Institute for Democracy and Economic Affairs | Think Tank | “Research institute dedicated to promoting solutions to public policy challenges.” | - Policy report |
| Institute of Economic Affairs | Think Tank | “Improve public understanding of the fundamental institutions of a free society, with particular reference to the role of markets in resolving economic and social problems.” | - Trade briefings |
| National Bureau of Economic Research | Think Tank | “Private, nonpartisan organization that facilitates cutting-edge investigation and analysis of major economic issues.” | - Pandemic preparedness report |
| Peterson Institute for International Economics | Think Tank | “Private, nonprofit, nonpartisan research institution devoted to the study of international economic policy.” | - Trade report |
| European Council on Foreign Relations | Think Tank | “Independent research on European foreign and security policy.” | - Power Atlas |
| OFSE (Austrian Foundation for Development Research) | Think Tank | “Research centre on questions of development policy and development cooperation.” | - Summary of existing access initiatives |
