# Supplementary material for: Transforming trade for vaccine equity: Policy gaps and barriers
Source: PLOS Glob Public Health. 2025 Jun 16;5(6):e0004012. doi: 10.1371/journal.pgph.0004012 (PMC12169585; doi:10.1371/journal.pgph.0004012)

**S3 Appendix:** PRISMA diagram showing search terms, titles screened and included, and reasons for exclusion.


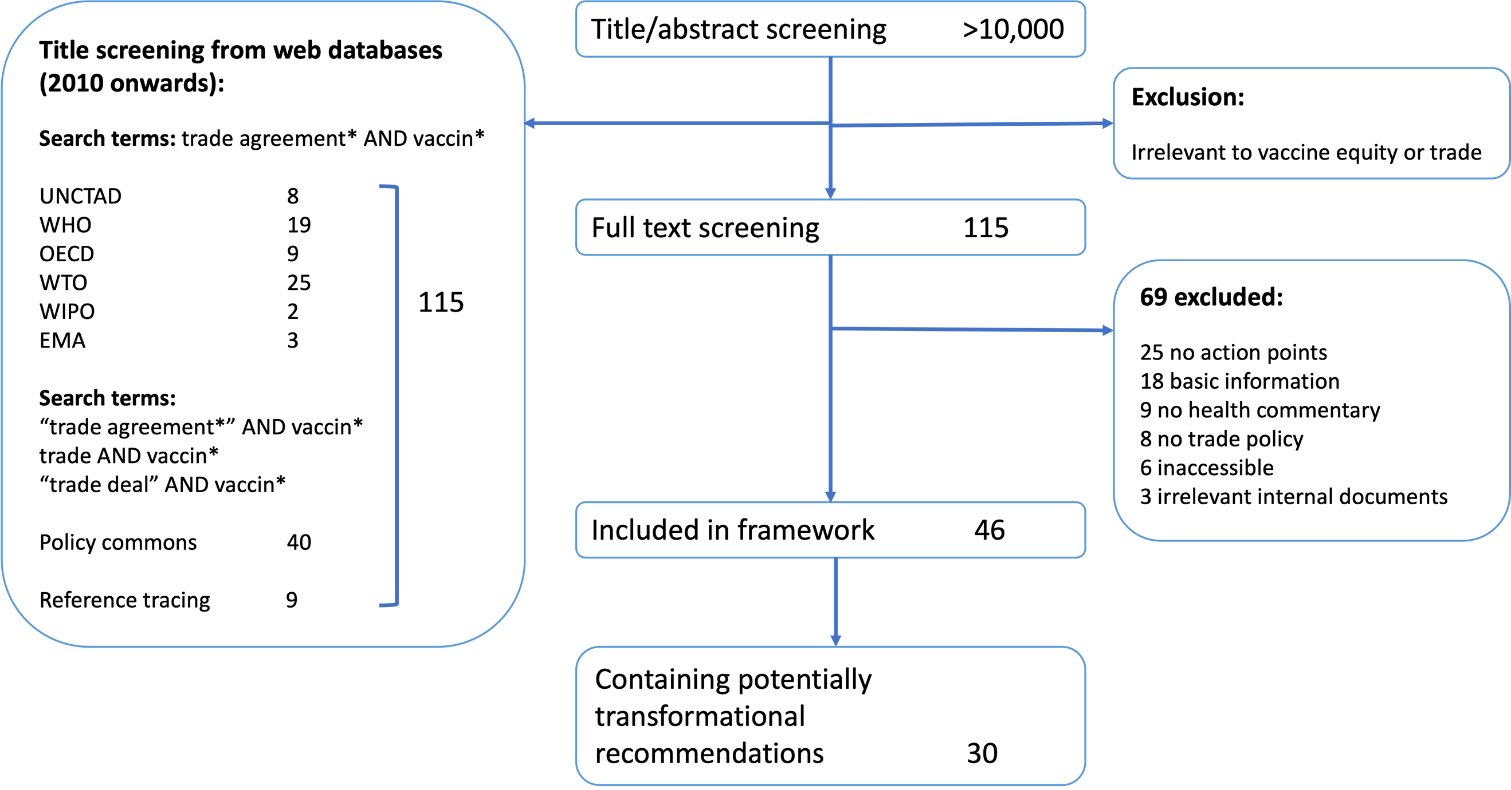

Supplement: S3 Appendix — (DOCX) [file pgph.0004012.s003.docx]
